# Evaluation of Mucin-producing Gallbladder Carcinoma Using Magnetic Resonance Cholangiopancreatography: A Case Report

**DOI:** 10.7759/cureus.4684

**Published:** 2019-05-17

**Authors:** Logan Burstiner, Bradley M White

**Affiliations:** 1 Radiology, Larkin Community Hospital, Miami, USA; 2 Interventional and Diagnostic Radiology, Larkin Community Hospital, Miami, USA

**Keywords:** mucin producing gallbladder carcinoma, mrcp

## Abstract

Mucin-producing gallbladder carcinoma (MPGBC) is a rare and aggressive subtype of gallbladder carcinoma. We present a case of MPGBC with associated magnetic resonance imaging (MRI) / magnetic resonance cholangiopancreatography (MRCP) findings that may raise suspicion of this diagnosis preoperatively.

## Introduction

In the United States, the incidence of gallbladder carcinoma is less than 5,000 cases per year [1]. Though this malignancy has several histological variants, mucin-producing gallbladder carcinoma (MPGBC) is a particularly rare and aggressive subtype. Recent studies suggest that it accounts for only 2.5%-5.5% of all gallbladder carcinomas [2-3].

MPGBC often presents with nonspecific, cholecystitis-like symptoms or is found incidentally during a cholecystectomy [4]. It can be difficult to differentiate from more benign entities using ultrasonography (US) or computed tomography (CT) alone [5-7]. As our case demonstrates, magnetic resonance imaging (MRI) / magnetic resonance cholangiopancreatography (MRCP) may be valuable in evaluating, and possibly diagnosing, MPGBC.

## Case presentation

**Figure 1:**
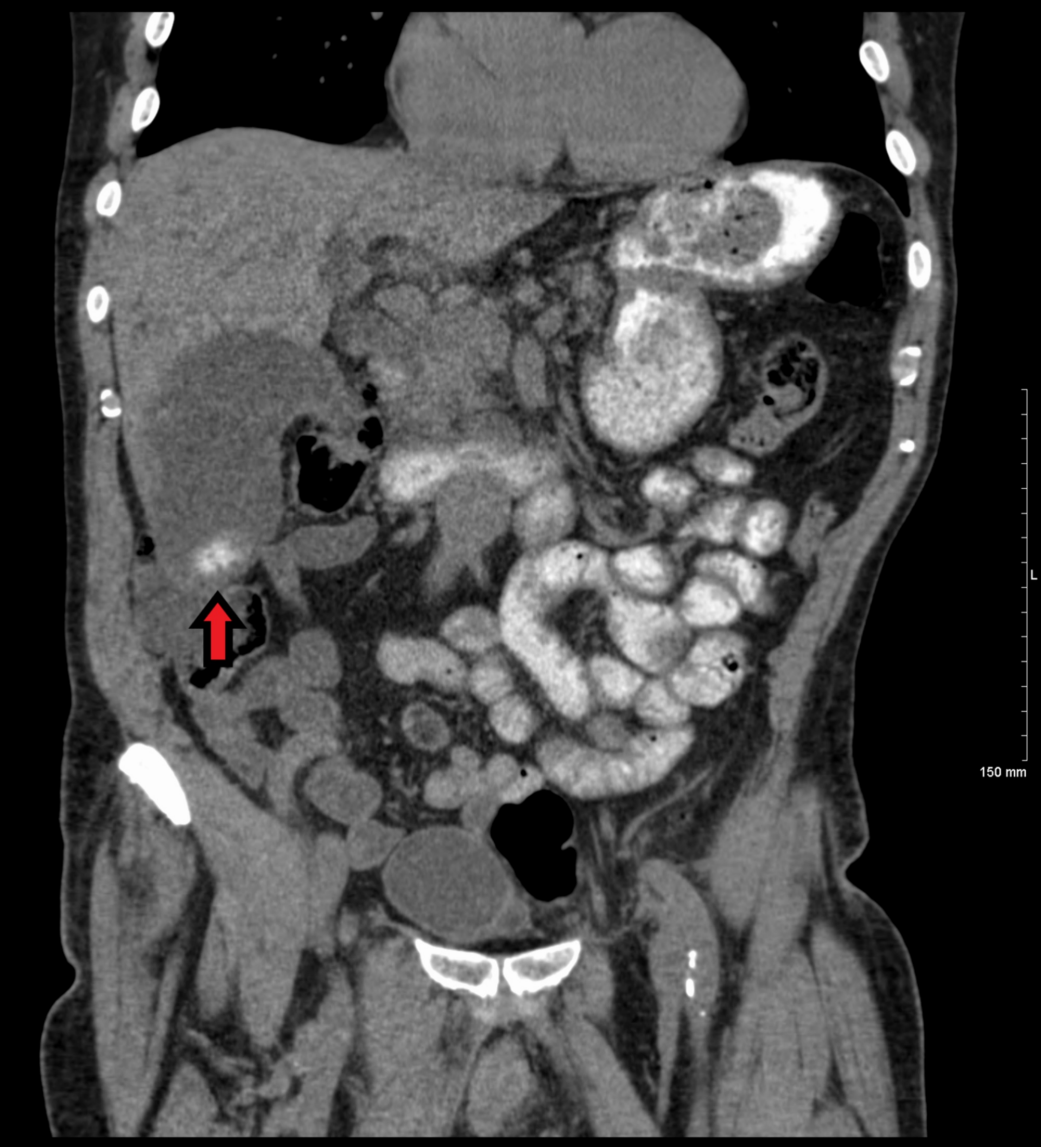
Coronal CT of the abdomen with oral contrast demonstrating a significantly dilated gallbladder. A 3 cm calcification is seen in the dependent portion of the fundus (arrow) CT - Computed Tomography

**Figure 2:**
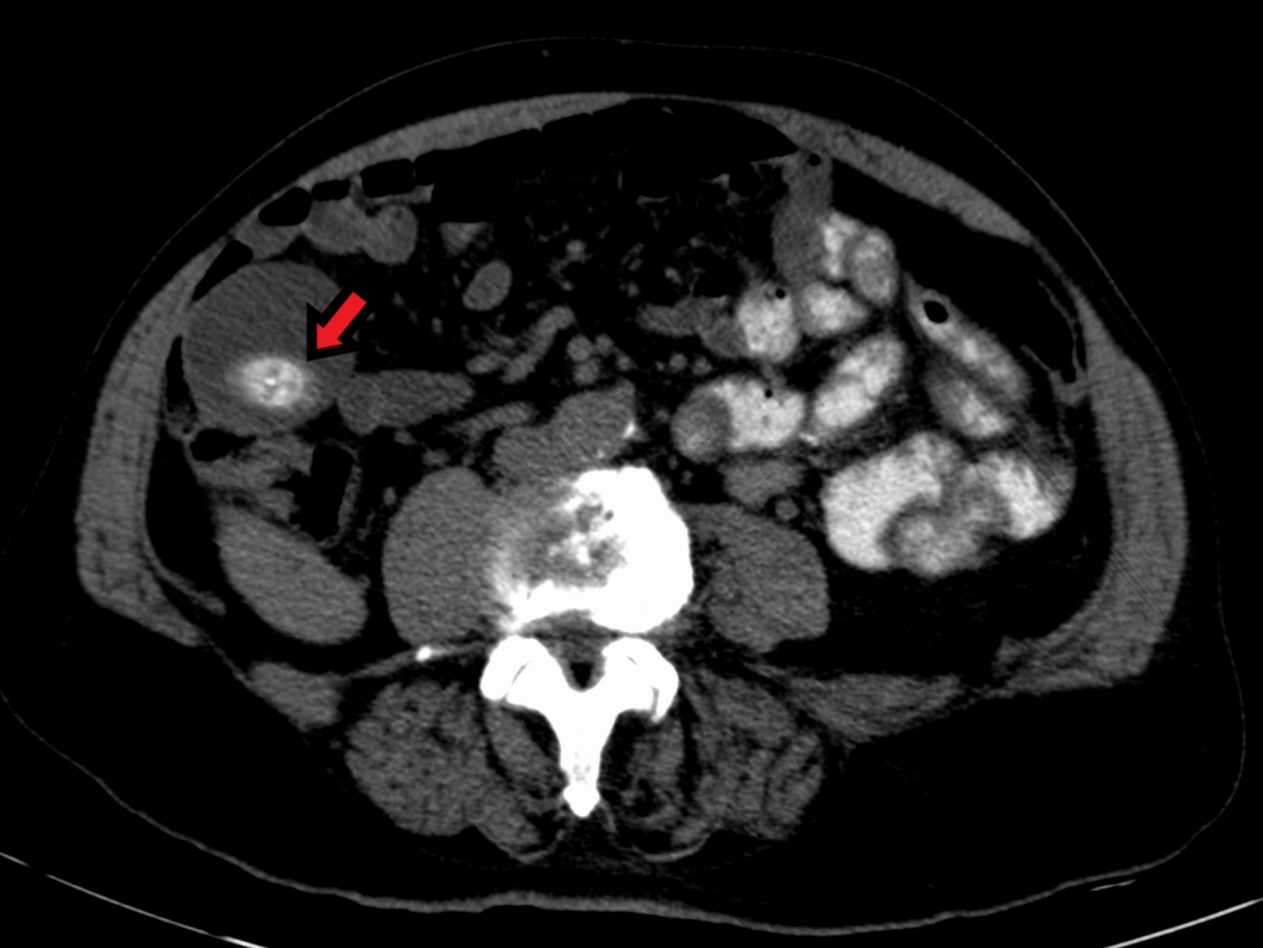
Axial view CT abdomen with oral contrast demonstrating a significantly dilated gallbladder. A 3 cm calcification is seen in the dependent portion of the fundus (arrow) CT - Computed tomography

**Figure 3:**
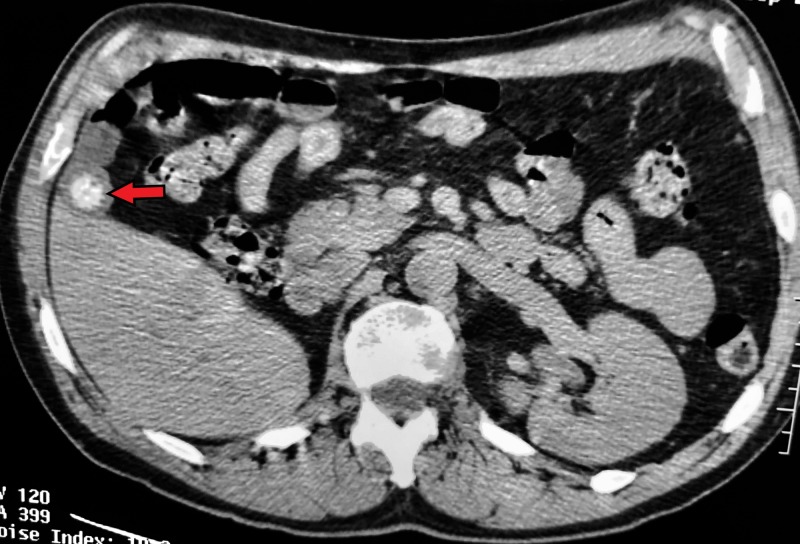
Axial CT of the abdomen without contrast taken 10 years prior. Prominent irregular calcification visible in the gallbladder fundus (arrow) CT - Computed tomography

We present a 78-year-old male with a past medical history of dementia, coronary artery disease, hypertension, and chronic obstructive pulmonary disease (COPD) who presented to the emergency department for evaluation of severe jaundice and itching. He denied abdominal pain. His hypertransaminemia and hyperbilirubinemia suggested obstructive jaundice. Abdominal CT with oral contrast was nonspecific, showing gallbladder dilation and what appeared to be a simple calcified gallstone in the dependent portion of the fundus (Figures 1-2). Notably, what was likely the same stone was seen on a CT abdomen performed 10 years prior, but further imaging was not done at that time (Figure 3).

**Figure 4:**
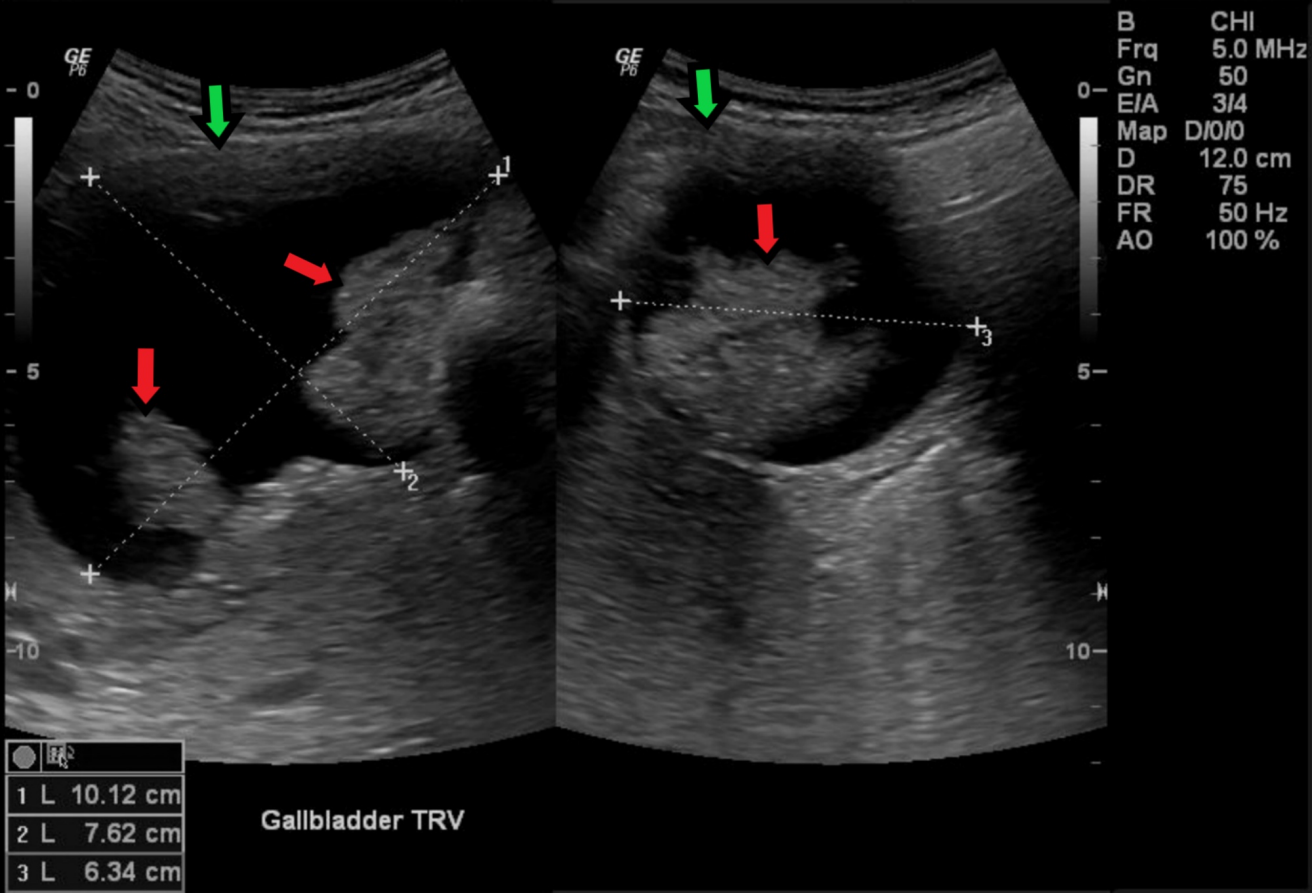
Transverse ultrasound of the gallbladder revealing multiple intraluminal masses (red arrows) and irregular wall thickening (green arrows). Gallbladder dimensions are given in the bottom left corner

**Figure 5:**
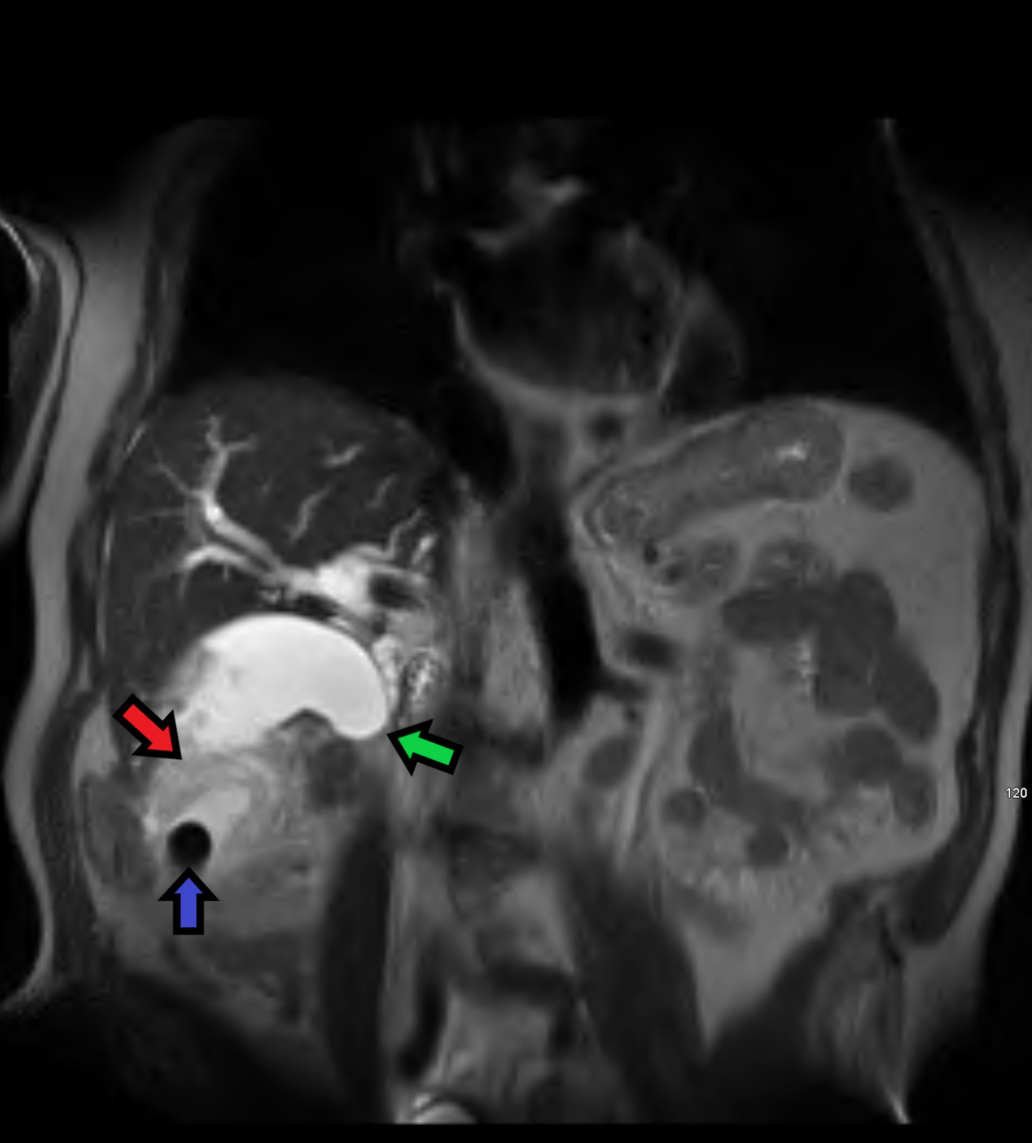
Coronal MRI T2 HASTE weighted image demonstrating a large mass (red arrow) surrounding a gallstone (blue arrow) in the distal portion of the gallbladder fundus. There is sharp tapering in the mid-to-distal common bile duct (green arrow) indicating obstruction secondary to infiltration by the mass MRI - Magnetic resonance image, HASTE - Half-Fourier Acquisition Single-shot Turbo Spin-Echo

**Figure 6:**
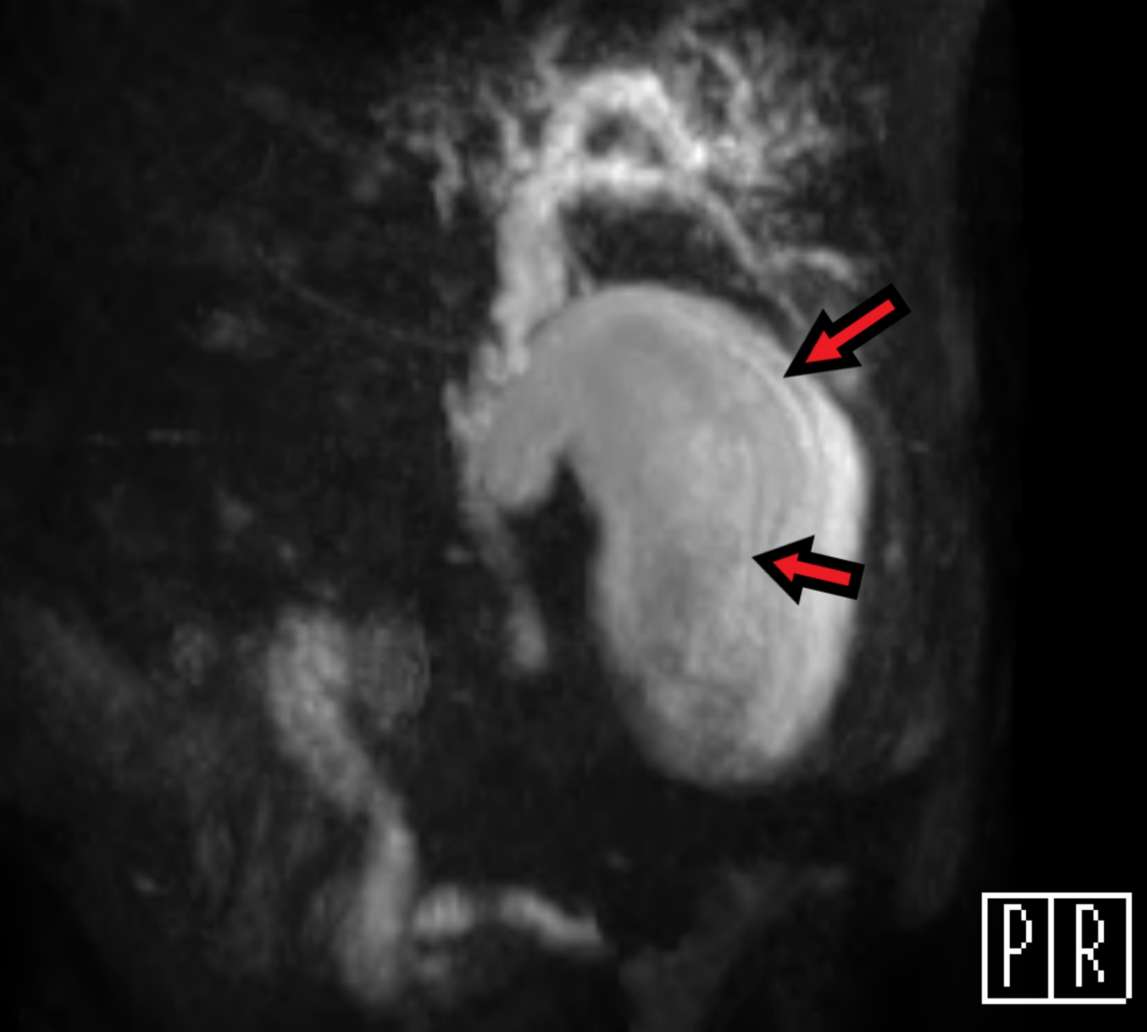
Oblique MRCP view of the gallbladder. Shows peripheral hypointense curvilinear striations parallel to the long axis (arrows) consistent with a mucous thread sign MRCP - Magnetic resonance cholangiopancreatography

During this evaluation, follow-up US revealed irregular intraluminal masses that raised suspicions for malignancy (Figure 4). Subsequent MRI/MRCP clearly demonstrated a large, irregular mass surrounding the gallstone. Of note, hypointense curvilinear striations were visible along the long axis of the gallbladder (Figures 5-6). This characteristic finding has been seen before and was coined a “mucous thread” sign. It has been theorized to be the result of excessive viscous mucin produced by this carcinoma [8].

**Figure 7:**
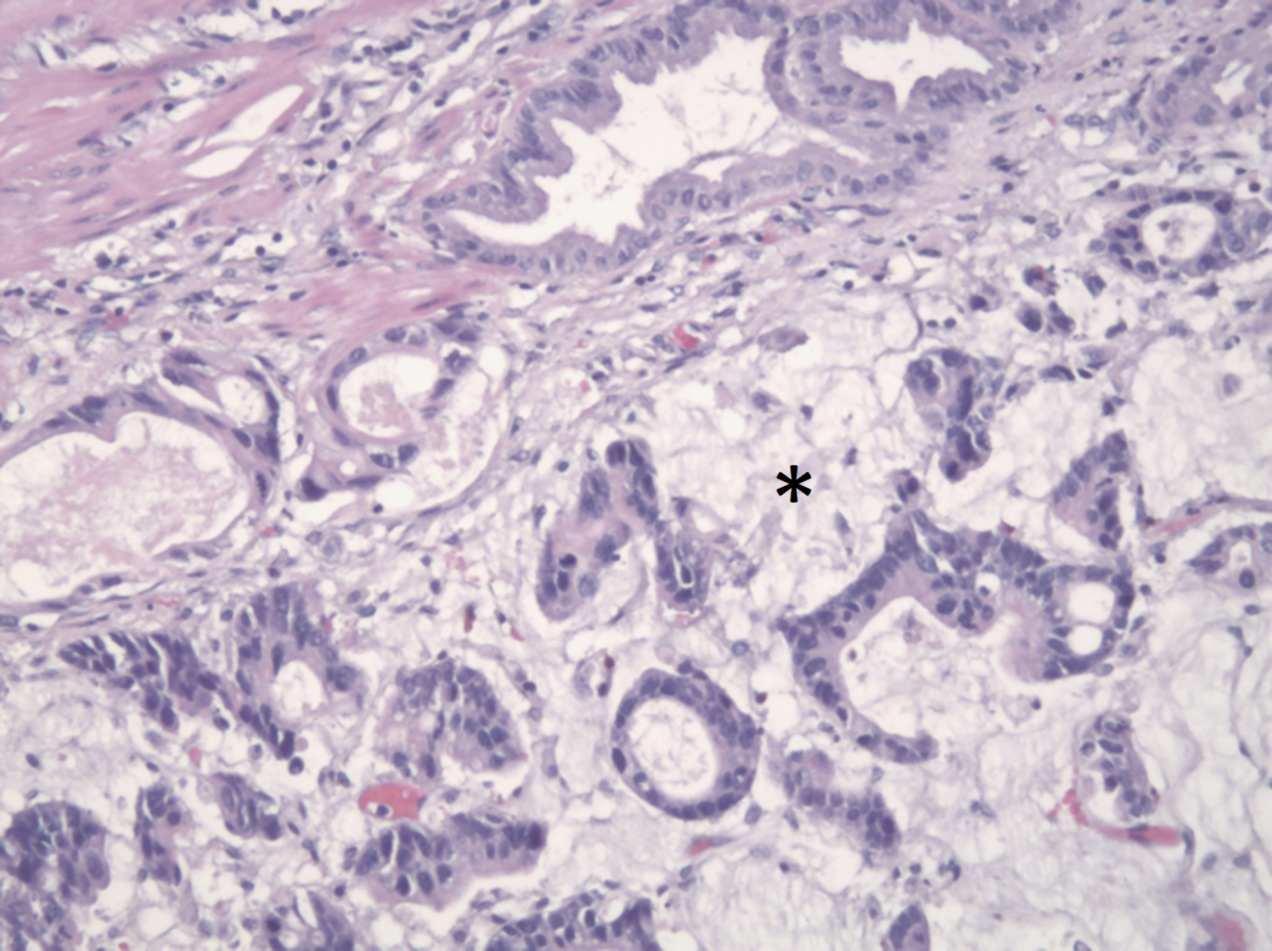
H&E stain showing malignant epithelial cells and glands surrounded by pools of extracellular mucin (asterisk), consistent with MPGBC H&E - Hematoxylin and Eosin, MPGBC - Mucin-producing gallbladder carcinoma

Though a laparoscopic cholecystectomy was performed, the area of invasion was too extensive for full resection. Histological samples confirmed MPGBC (Figure 7). The patient and his family declined further treatment of his malignancy, and he expired six months later.

## Discussion

MPGBC is extremely rare and can be overlooked or confused with less dangerous entities. It typically mimics acute cholecystitis and, therefore, most cases are diagnosed in the operating room during what is assumed to be a routine cholecystectomy. A careful imaging workup prior to surgery could inform clinicians and patients that they are dealing with a more malignant process. It is important for radiologists and other clinicians to be aware that the unique properties of this cancer may render some imaging modalities inferior to others.

Our case demonstrates the difficulty that CT can have in identifying MPGBC. Simply, a CT image is constructed based on the densities of different structures within the patient. Within the lumen of our patient's gallbladder, a hyperdense calcified gallstone is clearly visible. However, in these images, there is little evidence of a large mass surrounding the stone. Other researchers have described similar CT findings in cases of MPGBC. They have speculated that the excessive amount of mucin produced within the tumor may give it a density near to that of water; obscuring the mass by making it impossible to differentiate from fluid already within the gallbladder [7].

Ultrasonography is the initial imaging test of choice for any patient with possible gallbladder disease. Benefits include low cost and ease of use. In most cases, it adequately identifies masses in the gallbladder. However, it is less reliable in determining whether the mass is benign or malignant. It is inferior to other imaging modalities (CT and MRI) for identifying and staging gallbladder cancer. Due to this, current guidelines recommend following up any suspicious US findings with either a CT or an MRI [5-7].

As previously discussed, the MRI for our patient was much more informative than the other imaging techniques. While the CT could only visualize a gallstone, the MRI clearly depicted the large mass totally enveloping the stone and filling the gallbladder. At this point in the evaluation, a malignant process was obviously strongly suspected. Likely due to the rarity of MPGBC, it still came as a surprise when the intraoperative pathology report returned with this diagnosis.

Our retrospective investigation revealed that the MRCP may have suggested the lesion was this specific subtype of gallbladder cancer. It is possible that the hypointense striations along the long axis of the patient's gallbladder are unique radiographic signs of MPGBC. To our knowledge, there is no other case study discussing MRCP evaluation of MPGBC in United States literature. Japanese researchers, however, identified multiple patients with MPGBC who had similar linear hypointense areas visible on MRCP. They considered that the highly viscous properties of the mucin were responsible for the appearance of these characteristic striations and named this a “mucous thread” sign [8].

## Conclusions

Our case highlights the limitations of CT and US, as well as the value of MRI/MRCP, in the diagnostic evaluation of MPGBC. In addition, we draw attention to a unique radiographic finding of MPGBC (mucous thread sign) that may lead to the early detection of this highly fatal disease.
